# Differential genome-wide association analysis of schizophrenia and post-traumatic stress disorder identifies opposing effects at the *MAPT*/*CRHR1* locus

**DOI:** 10.3389/fgene.2026.1728494

**Published:** 2026-02-13

**Authors:** Zhong‐shan Cheng

**Affiliations:** Center for Applied Bioinformatics, St. Jude Children’s Research Hospital, Memphis, TN, United States

**Keywords:** *CRHR1*, differential effect size, GWAS, *MAPT*, PTSD, schizophrenia

## Abstract

**Background:**

Schizophrenia (SCZ) and post-traumatic stress disorder (PTSD) are severe psychiatric conditions with overlapping yet distinct symptomatology and pathophysiology.

**Methods:**

We conducted a differential genome-wide association study (GWAS) to directly compare the genetic architectures of SCZ and PTSD using publicly available GWAS summary statistics from the Psychiatric Genomics Consortium (PGC).

**Results:**

We identified four independent loci reaching genome-wide significance (P < 5 × 10^−8^) for differential associations between SCZ and PTSD in individuals of European ancestry. These loci (rs62062288, rs58120505, rs12536395, and rs11057189) showed genome-wide significant associations with SCZ and nominal associations with PTSD (all *P*s < 0.05) but with opposite directions of effect. Among these, only rs62062288 retained genome-wide significance for PTSD in trans-ancestry meta-analysis, whereas the other three loci showed markedly attenuated PTSD associations (all *P*s > 0.001). rs62062288 maps to the 17q21.31 *MAPT*/*CRHR1* locus, exhibiting a risk effect for PTSD and a protective effect for SCZ. GTEx analyses revealed that *MAPT* and *CRHR1* are highly expressed across multiple brain tissues and that rs62062288 shows opposite regulatory effects on *MAPT* and *CRHR1* expression in the brain tissue caudate basal ganglia, with the PTSD risk allele associated with increased *MAPT* expression and decreased *CRHR1* expression. Additional brain-region–specific expression quantitative trait locus (eQTL) effects on *CRHR1* were observed in frontal cortex BA9, hippocampus, and spinal cord, indicating tissue-dependent regulation.

**Conclusion:**

These findings provide evidence for distinct and opposing genetic contributions to SCZ and PTSD. The identification of the *MAPT*/*CRHR1* locus with brain-region–specific and gene-specific regulatory effects highlights divergent neurodevelopmental and stress-related pathways underlying these disorders.

## Introduction

Schizophrenia (SCZ) is one of the most severe and complex psychiatric disorders, affecting approximately 14–24 million individuals worldwide (Solmi et al., 2023). Its etiology reflects a multifactorial interplay between genetic susceptibility and environmental exposures. Genetic contributions to SCZ are well established, with heritability estimates ranging from 60% to 80%. The latest genome-wide association study (GWAS), including up to 76,755 SCZ cases and 243,649 controls, identified 287 loci significantly associated with the disorder (Trubetskoy et al., 2022), underscoring its highly polygenic architecture in which numerous variants of small effect collectively shape disease risk.

Beyond genetics, environmental factors play a critical role in SCZ onset and progression (Tosato et al., 2025). These include childhood trauma, substance abuse, urban upbringing, and exposure to acute or chronic stressors (Brown, 2011; Robinson and Bergen, 2021). Increasing evidence suggests that genetic vulnerability and environmental insults converge on shared biological pathways, including neuroinflammation, oxidative stress, and impaired neuroplasticity. For example, altered immune signaling and microglial activation have been implicated in aberrant synaptic pruning during neurodevelopment, a process strongly linked to SCZ pathophysiology (Sekar et al., 2016; Sellgren et al., 2019). Dysregulated oxidative stress responses may further compromise neuronal integrity and synaptic transmission, while deficits in neuroplasticity-related pathways disrupt learning, memory, and cognitive flexibility (Stephan et al., 2009). These interconnected mechanisms contribute to the marked clinical heterogeneity of SCZ and complicate the development of standardized treatment strategies, as patients with similar clinical presentations may arise from distinct etiological processes (Robinson and Bergen, 2021).

Post-traumatic stress disorder (PTSD) is another debilitating psychiatric condition that develops in a subset of individuals exposed to traumatic events, including warfare, natural disasters, interpersonal violence, or severe accidents (Stein et al., 2002). PTSD is characterized by heterogeneous symptom profiles encompassing intrusive memories, hyperarousal, avoidance behaviors, and persistent negative alterations in cognition and mood (Bryant et al., 2023). Like SCZ, PTSD arises from interactions between genetic predisposition and environmental stressors, although its heritability is more modest, estimated at approximately 30% (Duncan et al., 2018; Banerjee et al., 2017). Large-scale GWAS have demonstrated substantial shared genetic liability between PTSD and other psychiatric disorders, including SCZ, bipolar disorder, and major depressive disorder (Liberzon et al., 2017). The most recent PTSD GWAS, comprising 137,136 cases and 1,085,746 controls, identified 95 independent loci (Peyrot and Price, 2021), implicating biological processes related to stress reactivity, fear learning, immune regulation, and synaptic function.

Importantly, SCZ and PTSD share several core pathophysiological mechanisms that provide a strong biological rationale for comparative genetic analyses. Both disorders show evidence of neuroimmune dysregulation, including elevated inflammatory markers and altered microglial activity, suggesting immune-mediated effects on synaptic remodeling and neuronal signaling (Sekar et al., 2016; Peyrot and Price, 2021). Impaired neuroplasticity, reflected by disruptions in synaptic formation, maintenance, and elimination, has also been reported in both conditions and may underlie overlapping symptoms such as cognitive impairment and emotional dysregulation (Stephan et al., 2009; Pitman et al., 2012). In addition, oxidative stress and mitochondrial dysfunction have been implicated in both SCZ and PTSD, potentially contributing to long-term neural circuit instability following genetic or environmental insults (Ng et al., 2008).

Despite these shared mechanisms, substantial biological divergence exists between the two disorders. SCZ is more strongly linked to early neurodevelopmental abnormalities, widespread synaptic pruning deficits, and dysregulation of dopaminergic and glutamatergic neurotransmission (Sellgren et al., 2019; Owen et al., 2016). In contrast, PTSD is more closely associated with trauma-specific alterations in fear conditioning and extinction, amygdala–prefrontal circuitry dysfunction, and sustained dysregulation of the hypothalamic–pituitary–adrenal (HPA) axis (Liberzon and Abelson, 2016; Yehuda and LeDoux, 2007). These distinctions suggest that while overlapping genetic factors may influence general vulnerability to psychopathology, disorder-specific genetic variants likely modulate distinct molecular pathways and neural circuits.

Although GWAS have successfully identified loci shared between SCZ and PTSD, most studies have focused on genetic correlation rather than directly contrasting genetic effects between disorders. Differential GWAS approaches (Peyrot and Price, 2021; Cheng et al., 2023), which explicitly test for differences in effect size and direction across traits, provide powerful frameworks to disentangle shared versus disorder-specific genetic architectures. To address this gap, we leveraged large-scale GWAS summary statistics for SCZ and PTSD from the Psychiatric Genomics Consortium (PGC) to perform a differential GWAS analysis. This approach prioritized four genome-wide significant loci exhibiting differential associations with opposite effect directions between the two disorders and further confirmed the loci *MAPT*/*CRHR1* represented by rs62062288, which is genome-wide significantly associated with PTSD in a trans-ancestry GWAS, offering novel insights into genetic factors that may drive the shared and distinct pathophysiological pathways between SCZ and PTSD.

## Materials and methods

### GWAS summary statistics

We utilized publicly available GWAS summary statistics for SCZ and PTSD from the PGC. The SCZ GWAS (PGC3_SCZ_wave3.european.autosome.public.v3.vcf.tsv.gz) dataset encompasses European ancestry individuals, and the PTSD GWAS (eur_ptsdcasecontrol_pcs_v4_aug3_2021.vcf.gz) similarly consists of individuals of European descent. Both datasets were downloaded from the PGC website (https://www.med.unc.edu/pgc/download-results/). Trans-ancestry GWAS of PTSD among samples with European, African, and Hispanic ancestries were conducted by PGC with a quantitative phenotype of PTSD, in contrast to the binary phenotypes of SCZ and PTSD used for the aforementioned GWASs of the two traits by PGC. GWAS summary statistics of trans-ancestry PTSD was used to evaluate candidate SNPs showing differential associations between SCZ and PTSD, in an effort of confirming these SNPs’s associations with PTSD among multiple populations.

### Data preprocessing

All analyses were performed within SAS Studio provided by the free online cloud-based SAS OnDemand for Academics (https://www.sas.com/en_us/software/on-demand-for-academics.html). GWAS summary files were uncompressed and imported directly using the custom macro %ImportFileHeadersFromZIP provided by the COVID-19_GWAS_Analyzer (https://github.com/chengzhongshan/COVID19_GWAS_Analyzer) (Zhang et al., 2024), which parses the necessary columns. For SCZ, the variables retained including chromosome, SNP identifier, position, alternative allele, non-effect allele, effect size, standard error, *p*-value, and other relevant metrics. For PTSD, the corresponding variables were similarly extracted.

Only PTSD SNPs with minor allele frequency ≥0.05 were retained, and SNPs were harmonized between the two GWASs, and SNPs were aligned similarly so that the effect allele corresponded across both datasets. In cases of SNPs with its alleles flipped, the effect sizes of these SNPs for SCZ were reversed and alleles of these SNPs were swapped to match with that from PTSD. SNPs where matching was impossible based on genomic position and two alleles were excluded. Only SNPs present in both summary statistics, with matching alleles at identical genomic positions, were retained for downstream analysis.

### Differential GWAS analysis

To identify loci with differential genetic effects between SCZ and PTSD, we applied the SAS macro %DiffTwoGWAS from COVID-19_GWAS_Analyzer according to (Cheng et al., 2023) with update adjusting the potential sample overlap between two tested GWASs as described in the following. In details, we initially followed the traditional method to determine differential effect sizes by adjusting the different effect sizes of each SNP using estimated variance based on the square root of the sum of standard errors of each SNP from the two GWASs (Cheng et al., 2023; Luo et al., 2022; Thibord et al., 2022). To address potential effect of overlapped samples on the variance, it is necessary to estimate correlation (ρ) of SNPs with low linkage disequilibrium (LD R^2^ < 0.1) in a specific population in line with the samples used in the two GWASs, and then subtracting the 2*ρ*se1*se2 from the variance when estimating the delta-zscore. The rational for the estimation is detailed as follows:

Under standard large-sample theory, differential effect size is estimated using the formula: $Z=\left(\beta 1-\beta 2\right)/sqrt\left(se1^{2}+se2^{2}\right)$ , in which the reported standard errors (se1 and se2 for each SNP in the two GWASs) already encodes the sample size and sampling variability. So if no sample overlap between two GWASs, there would be no need for extra adjustment for the formula for estimating differential effect size of each SNP.

When there are partial or full sample overlap between two GWASs, these effect sizes for each SNP between the two GWASs would be correlated. In this situation, it is necessary to adjust the variance used to calculate differential effect size of each SNP. The theory is this: Var(β1 – β2) = Var(β1) + Var(β2) – 2*Cov(β1, β2) = se1^2^ + se2^2^–2* Cov(β1, β2). A practical way to estimate Cov(β1, β2) is to use lower LD SNPs (R^2^ < 0.1) and its corresponding effect sizes in the two GWASs, which is helpful when the raw genotyping data of two GWASs are not accessible. The correlation of z-scores provides an empirical correlation ρ genome-wide and can be plugged with se1 and se2 into the formula used to estimate differential effect sizes as Var(β1 – β2) = se1^2^ + se2^2^–2*ρ*se1*se2.

To estimate the statistical significance for the delta-zscore based on the above updated formula, it is necessary to restrict mean of delta-zscore to be 0 and its corresponding variance equal to 1. This is especially important when the empirical estimation provided in the above situation fails to normalize the biases between two GWASs especially when either or both of the two GWASs including too many genome-wide significant SNPs. So the following procedure is proposed to generate statistical significance for comparing SNP effect sizes on genome-wide:

It would be more meaningful to consider SNPs with both nominal significant association with opposite effect sizes in the two GWASs, requiring these SNPs also show significant differential associations between the two GWASs. This is especially important for further reducing the biases when one SNP has extreme significance in one GWAS but shows no significance in the other GWAS; as this SNPs is genome-wide significant in the original GWAS and has been reported by previous researchers, as well as it is not associated with the other trait, there would be no biological mechanisms for the SNP associated with both traits. Thus, it is necessary to only focus on SNPs showing genome-wide significantly differential effect sizes (*P* < 5E-8) and also show nominal significance (*P* < 0.05) but with opposite effect sizes in both GWASs.

### Gene expression analysis and expression quantitative trait locus (eQTL) analysis

COVID-19_GWAS_Analyzer was utilized to perform analyses in this section. The SAS macro %Boxplots4GenesInGTExV8ByGrps was applied to evaluate genes adjacent to rs62062288 using data from the Genotype-Tissue Expression database (GTEx) (GTEx Consortium, 2020). Two SAS macros, including %Query_GTEx_eQTLs4SNP and %heatmap4longformatdsd, were used to make eQTL association and effect size heatmap; the SAS macro %CaculateMulteQTLs_in_GTEx was used to make eQTL boxplot for rs62062288 among GTEx brain tissues. All procedures were followed based on the previously published protocol (Zhang et al., 2024).

## Results

### Differential GWAS between SCZ and PTSD

**FIGURE 1:**
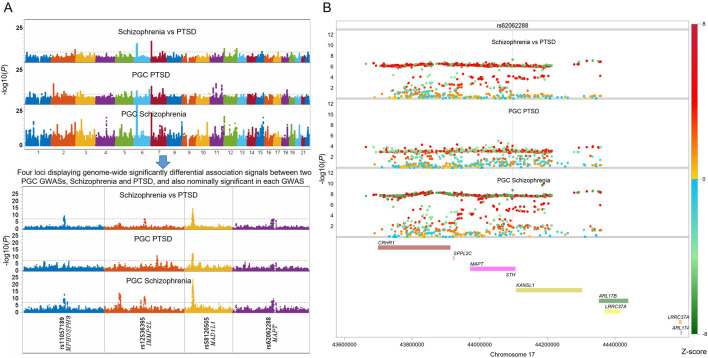
Differential genome-wide association analysis between Schizophrenia (SCZ) and Post-Traumatic Stress Disorder (PTSD) identifies four loci with opposite associations with both traits. **(A)** Manhattan plots showing genome-wide significant associations from the PGC GWASs of SCZ and PTSD, as well as the differential GWAS comparing the two traits. Four loci with genome-wide significantly differential associations and also nominally associated with both traits in different effect directions are highlighted below the plots, represented by the leading SNPs and their nearby genes. **(B)** One of these leading SNP with the differential associations is rs62062288, located near *MAPT*. Local Manhattan plots show the association signals in each of the two GWASs and in the differential analysis. SNPs with positive and negative differential z-scores are colored using different color schemes. All GWAS signals are truncated at –log_10_(*P*) = 30 for better visualization. Similar local Manhattan plots for other three top hits are included in the Supplementary Figures S1-S3.

To identify genomic loci differentially associated with SCZ and PTSD, we performed a differential GWAS by directly estimating potential sample overlap using pruned SNPs (R^2^ < 0.1), contrasting GWAS summary-level data from large-scale PGC studies of these two disorders, and calculate adjusted differential effect sizes as well as its corresponding differential association *p*-values. Manhattan plots of the individual GWAS for SCZ (cases = 76,755, controls = 243,649) and PTSD (cases = 137,136, controls = 1,085,746), as well as the differential GWAS, are shown in Figure 1A.

The differential GWAS revealed four independent loci that exceeded genome-wide significance (*P* < 5 × 10^−8^) for differences in associations between SCZ and PTSD after adjusting sample overlap, with both passed nominal significance of *P* < 0.05 in each GWAS but displaying opposite effect sizes. These loci are highlighted in Table 1, which lists out their lead SNPs, chromosomal positions, nearest genes, effect sizes, standard errors, and *p*-values in the differential GWAS, as well as their respective GWAS statistics in both SCZ and PTSD.

**TABLE 1:** Four loci showing genome-wide significantly differential associations between Schizophrenia (SCZ) and Post-Traumatic Stress Disorder (PTSD).

Chr	Rsid	A1	A2	Gene	SCZ association			PTSD association			SCZ vs. PTSD	
					Beta	SE	*P*	Beta	SE	*P*	Diff zscore	Diff *P*
17	rs62062288	A	G	*MAPT*	−0.068	0.011	1.65E-09	0.023	0.006	7.14E-05	−5.46	4.87E-08
12	rs11057189	T	G	*MPHOSPH9*	0.073	0.010	5.67E-14	−0.019	0.005	4.30E-04	6.33	2.42E-10
7	rs58120505	T	C	*MAD1L1*	0.090	0.009	2.24E-24	−0.013	0.004	1.70E-03	7.99	1.30E-15
7	rs12536395	A	G	*IMMP2L*	−0.063	0.009	1.13E-11	0.013	0.005	3.49E-03	−5.58	2.40E-08

Abbreviations are Chr: chromosomal, Rsid: SNP identification id, A1: alternative allele, A2: reference allele, Diff: differential GWAS between SCZ GWAS and PTSD GWAS. Only SNPs with both opposite and nominally significant associations with SCZ and PTSD are displayed in the table. The two GWASs, were conducted using the case-control design.

Notably, one of the SNPs with the differential signals demonstrating genome-wide significant association with SCZ and also displaying suggestive significance with PTSD was rs62062288 (located near *MAPT* on chromosome 17), with a corresponding differential *p*-value of 4.87 × 10^−8^. As illustrated in Figure 1B, this region showed opposing directions of association in the two disorders: while rs62062288 was genome-wide significantly associated with SCZ showing protective effect, it tends to be a risk SNP to PTSD, although the SNP is only nominally significantly associated with PTSD. Further evaluation of the SNPs in trans-ancestry GWAS of quantitative PTSD confirmed that it was genome-wide significantly associated with quantitative PTSD score as a risk marker (see Table 2). This SNP is highly linked with a known PTSD risk SNP rs2532252 (R^2^ = 0.81 in European population), which was reported to be genome-wide significantly associated with PTSD sub-symptom re-experience in the Million Veteran Program (MVP) cohort and was replicated in UK Biobank cohort (Gelernter et al., 2019). The local Manhattan plot (Figure 1B) around rs62062288 further highlights the specificity and robustness of this SNP, with neighboring SNPs demonstrating similar trends.

**TABLE 2:** Trans-ancestry PTSD associations for four loci showing genome-wide significantly differential associations between Schizophrenia (SCZ) and Post-Traumatic Stress Disorder (PTSD).

Chr	Rsid	A1	A2	Z	*P*
17	rs62062288	A	G	5.631	1.79E-08
12	rs11057189	T	G	−1.675	0.09403
7	rs58120505	T	C	−3.224	0.001264
7	rs12536395	A	G	1.557	0.1195

Abbreviations are Chr: chromosomal, Rsid: SNP, identification id, A1: alternative allele, A2: reference allele, Z: meta-analysis zscore, and *P*: meta-analysis *p*-value. The association results for the 4 SNPs, were extracted from quantitative PTSD, trans-ancestry GWAS, published by (Nievergelt et al., 2024).

Other prominent loci exhibiting genome-wide significantly differential association included variants rs11057189 (*MPHOSPH9*), rs58120505 (*MAD1L1*), and rs12536395 (*IMMP2L*) (see Table 1; Supplementary Figures S1-S3). However, further searching for these SNPs in the trans-ancestry quantitative PTSD GWAS revealed that no SNPs, except rs62062288, displayed robust association with quantitative PTSD score, with no SNPs showing significance of *P*s > 0.001 (see Table 2). Thus, we excluded them in downstream analysis.

Taken together, our results indicate that SCZ and PTSD share some polygenic risk, with rs62062288 representing a locus showing strong evidence for differential genetic association between the two disorders, implicating biological processes that may be uniquely relevant to the etiology or pathophysiology of each disorder.

### Potential underlying mechanism for the opposite associations of rs62062288 with SCZ and PTSD

We suspected that rs62062288 may regulate its adjacent genes and consequently influence the predisposition/protection to PTSD and SCZ, respectively. To characterize the regulatory landscape surrounding rs62062288 at the 17q21.31 locus, we examined gene expression patterns and expression quantitative trait locus (eQTL) effects using GTEx data. Across 54 tissues, eight genes located near rs62062288 (*CRHR1*, *SPPL2C*, *MAPT*, *KANSL1*, *ARL17A*, *ARL17B*, *LRRC37A*/*LRRC37A2*) showed variable expression profiles, with *CRHR1* and *MAPT* exhibiting relatively high expression across multiple brain tissues compared with the other genes in the region (Supplementary Figure S4A).

eQTL analysis demonstrated that rs62062288 was significantly associated with the expression of all eight genes in at least one GTEx tissue (Supplementary Figure S4B). Notably, in the caudate basal ganglia, rs62062288 showed opposite directions of association with *CRHR1* and *MAPT* expression. The minor allele (A) was associated with lower *CRHR1* expression and higher *MAPT* expression in this brain region.

Further examination of brain-specific eQTL effects revealed that rs62062288 was also significantly associated with *CRHR1* expression in additional brain tissues, including frontal cortex BA9, hippocampus, and spinal cord cervical c-1 (Supplementary Figures S4B and S4C). The direction of effect varied across these regions, with a positive association observed in frontal cortex BA9 and negative associations observed in the hippocampus, spinal cord cervical c-1, and caudate basal ganglia, indicating tissue-dependent regulatory effects of rs62062288 on *CRHR1* expression.

Together, these results demonstrate that rs62062288 exerts gene- and brain-region–specific regulatory effects at the 17q21.31 locus, including opposing regulation of *CRHR1* and *MAPT* in the caudate basal ganglia.

## Discussion

In this study, we leveraged large-scale GWAS datasets to perform a direct comparison of the genetic architectures of SCZ and PTSD. Our differential GWAS approach enabled the identification of four independent loci that exhibit genome-wide significant differences in their associations with these two psychiatric disorders, although only one SNP rs62062288 was prirotized based on a trans-ancestry PTSD GWAS using quantitative PTSD score as phenotype. rs62062288 is located at the 17q21.31 locus as a marker representing the locus covering two important genes, including *MAPT* and *CRHR1*, and the locus exhibiting opposite genetic effects on SCZ and PTSD, with its minor allele conferring protection against SCZ simultaneously increasing risk for PTSD.

rs62062288 representing the locus covering *MAPT*/*CRHR1* shows opposing associations with PTSD and SCZ, consistent with antagonistic pleiotropy (Bian et al., 2024). Our analyses suggest that these divergent effects are mediated through distinct but complementary biological pathways involving *CRHR1* and *MAPT*, reflecting fundamental differences between stress-related and neurodevelopmental disease mechanisms, that are specifically associated with PTSD and SCZ.

Expression profiling across GTEx tissues (GTEx Consortium, 2015) demonstrates that *CRHR1* and *MAPT* are highly expressed in multiple brain regions, supporting their relevance to psychiatric phenotypes (Supplementary Figure S4A). eQTL analysis shows that rs62062288 is associated with all eight nearby genes in at least one tissue, but exhibits opposite regulatory effects on *CRHR1* and *MAPT* in the caudate basal ganglia (Supplementary Figure S4B). Specifically, the rs62062288*A allele is associated with reduced *CRHR1* expression and increased *MAPT* expression in this region, highlighting the caudate as a key site where stress-related and neurodevelopmental pathways may diverge.

In the context of SCZ, altered neurodevelopmental trajectories and synaptic dysfunction are central features of disease pathophysiology (Sekar et al., 2016; Sellgren et al., 2019). *MAPT* encodes the microtubule-associated protein tau, a key regulator of microtubule stability, axonal transport, and synaptic integrity (Mom et al., 2010; Avila et al., 2004). Beyond its role in neurodegeneration, tau-related pathways are increasingly recognized as important for neuronal maturation, synaptic pruning, and circuit-level organization (Rissman, 2009). Genetic variants such as rs62062288 that promote *MAPT* expression or support tau-mediated cytoskeletal stability may therefore confer resilience to neurodevelopmental disruptions that predispose to SCZ, consistent with evidence linking synaptic pruning and connectivity abnormalities to SCZ risk (Schizophrenia Working Group of the Psychiatric Genomics Consortium, 2014).

In contrast, PTSD is conceptualized as a stress-related disorder characterized by maladaptive fear learning, impaired extinction, and persistent alterations in stress-responsive neural circuits, particularly within the amygdala, hippocampus, and prefrontal cortex (Banerjee et al., 2017). *CRHR1* plays a central role in HPA‐axis signaling and stress responses, and genetic variation in *CRHR1* has been repeatedly associated with PTSD risk and symptom severity following trauma exposure (Gelernter et al., 2019; Nievergelt et al., 2024). The reduced *CRHR1* expression associated with rs62062288 in the caudate and hippocampus, together with region-specific effects in frontal cortex BA9 and spinal cord (Supplementary Figure S4C), suggests context-dependent dysregulation of stress signaling.

Importantly, tau encoded by *MAPT* is dynamically regulated by neuronal activity and stress-related signaling pathways. Both acute and chronic stress have been shown to induce tau phosphorylation and mislocalization in limbic brain regions (Rissman, 2009; Alvarez-de-la-Rosa et al., 2005; Regan et al., 2015; Sotiropoulos et al., 2011), processes implicated in maladaptive synaptic remodeling. *MAPT* variants that increase tau expression or alter its regulation may therefore heighten vulnerability to stress-induced plasticity, amplifying maladaptive circuit changes following trauma exposure. In this framework, genetic variants that stabilize neurodevelopmental processes may paradoxically increase PTSD risk by enhancing stress-sensitive plasticity mechanisms in fear and memory circuits.

Together, these findings identify *CRHR1*-and *MAPT*-mediated pathways as converging but functionally distinct mechanisms through which rs62062288 influences psychiatric risk. *CRHR1*-related effects primarily implicate stress-response and HPA-axis dysregulation relevant to PTSD, whereas *MAPT*-related effects implicate neurodevelopmental and synaptic pathways relevant to SCZ. This context-dependent genetic architecture underscores the importance of developmental timing, environmental exposure, and brain-region specificity when interpreting pleiotropic genetic associations.

Several limitations should be noted. The current analyses are based on statistical associations derived from publicly available GWASs, transcriptomics and eQTL datasets and lack direct experimental validation, precluding definitive causal inference; in terms of differential GWAS, it only exclusively focused on individuals of European ancestry, which may limit generalizability to other populations. Our differential GWAS was performed by considering potential sample overlap as we indeed found the two PGC GWASs displayed sample overlap based on pruned low linkage disequilibrium SNPs (R^2^ < 0.1). One potential issue is related to whether these overlapped samples having comorbidity between PTSD and SCZ were excluded in the individual GWAS of PTSD or SCZ, which may potentially affect the final top hits in the differential GWAS. Another potential limitation about the differential GWAS is that if either of the two GWASs has false significant association signals that were not due to real biology but technique errors, such as inappropriate quality control for genotypes and phenotype when performing GWAS, current method cannot exclude them. Only further replication with independent cohorts can determine real SNPs showing differential effect sizes between the two target traits. This is why we used trans-ancestry GWAS of PTSD to further prioritize these top hits, as SNPs with both strong associations but opposite effects with SCZ and PTSD would be more reliable. In addition, most GTEx samples are derived from non-diseased adult tissues and may not capture developmental or trauma-dependent regulatory effects. Future studies integrating functional experiments, brain-region–and cell-type–specific transcriptomics, and sex-stratified analyses will be essential to validate the proposed mechanisms linking *CRHR1* and *MAPT* to divergent risk for PTSD and SCZ.

## Data Availability

The original contributions presented in the study are included in the article/Supplementary Material, further inquiries can be directed to the corresponding author.

## Supplementary material

SUPPLEMENTARY FIGURE S1 Local Manhattan plots showing rs11057189 with genome-wide significantly differential associations between PGC GWASs of SCZ and PTSD. The effect size directions, including negative and positive association for these SNPs with two traits, are colored used different schemes, respectively, in the plots.

SUPPLEMENTARY FIGURE S2 Local Manhattan plots showing rs58120505 with genome-wide significantly differential associations between PGC GWASs of SCZ and PTSD. The effect size directions, including negative and positive association for these SNPs with two traits, are colored used different schemes, respectively, in the plots.

SUPPLEMENTARY FIGURE S3 Local Manhattan plots showing rs12536395 with genome-wide significantly differential associations between PGC GWASs of SCZ and PTSD. The effect size directions, including negative and positive association for these SNPs with two traits, are colored used different schemes, respectively, in the plots.

SUPPLEMENTARY FIGURE S4 Brain-region–specific regulatory effects of rs62062288 on *CRHR1 *and *MAPT* expression. **(A)** Median gene expression levels across 54 GTEx tissues for 8 genes located near rs62062288 (*CRHR1, SPPL2C, MAPT, KANSL1, ARL17A, ARL17B, LRRC37A/LRRC37A2*). Among these genes, *CRHR1* and *MAPT* show relatively high expression across multiple brain tissues, supporting their relevance to central nervous system function. **(B)** Expression quantitative trait locus (eQTL) analysis of rs62062288 across GTEx tissues. The SNP is significantly associated with the expression of all 8 genes in at least one tissue. Notably, in the caudate basal ganglia, the rs62062288 minor allele A shows opposite regulatory effects, being associated with decreased *CRHR1* expression and increased *MAPT* expression, highlighting a potential mechanism for divergent psychiatric associations. **(C)** Brain-specific eQTL effects of rs62062288 on *CRHR1* expression across multiple regions, including frontal cortex BA9, hippocampus, spinal cord cervical c-1, and caudate basal ganglia. The direction of association varies by brain region, indicating tissue-dependent regulation of stress-related signaling pathways. Together, these results implicate the caudate basal ganglia as a key anatomical site where rs62062288 may differentially influence stress-related (PTSD) and neurodevelopmental (Schizophrenia) pathways through *CRHR1* and *MAPT*.
