# Supplementary figures and images for: Differential genome-wide association analysis of schizophrenia and post-traumatic stress disorder identifies opposing effects at the MAPT/CRHR1 locus
Source: Front Genet. 2026 Feb 13;17:1728494. doi: 10.3389/fgene.2026.1728494 (PMC12945334; doi:10.3389/fgene.2026.1728494)

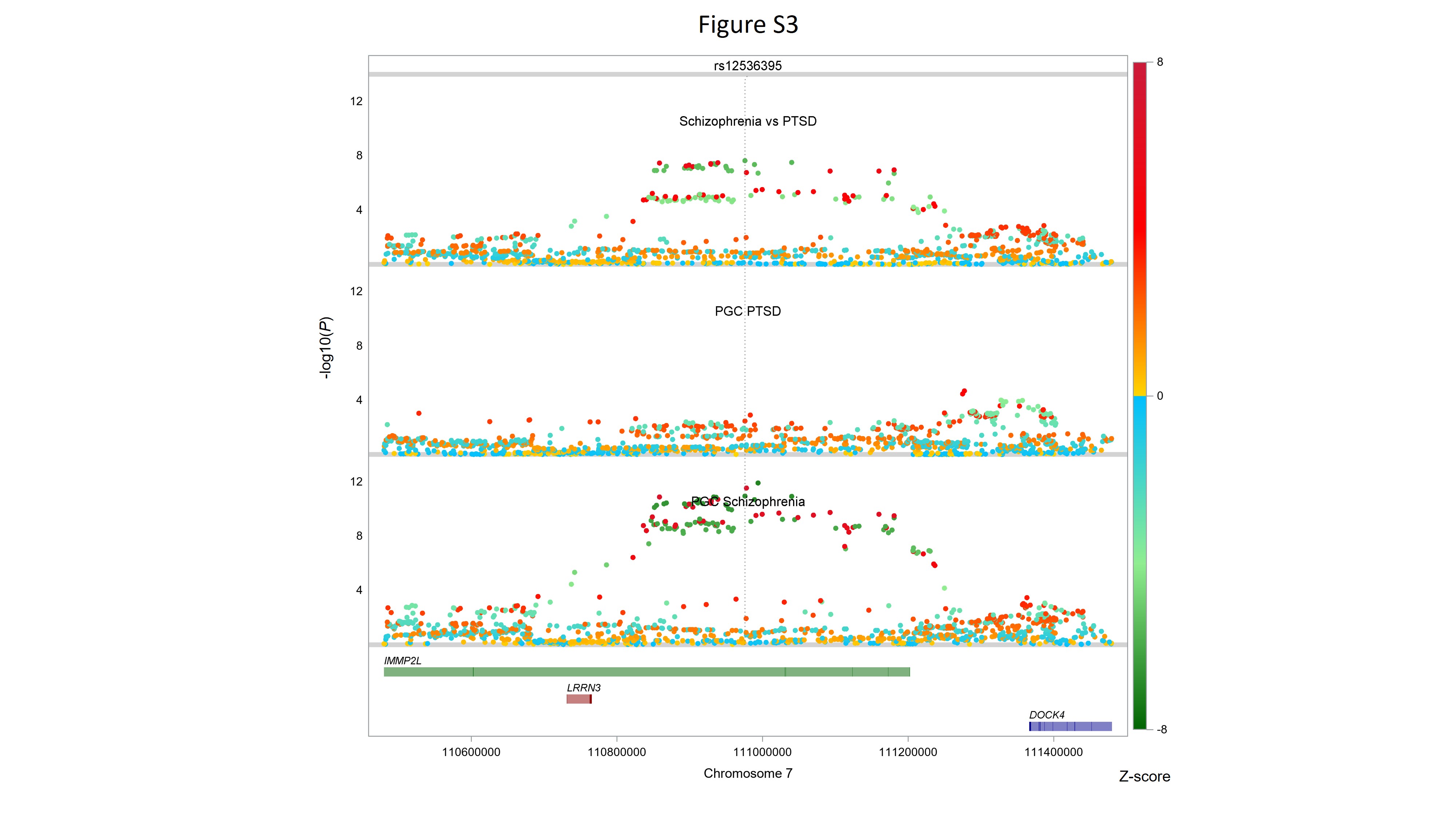

Supplement: Supplementary file 1 [file Image3.jpeg]

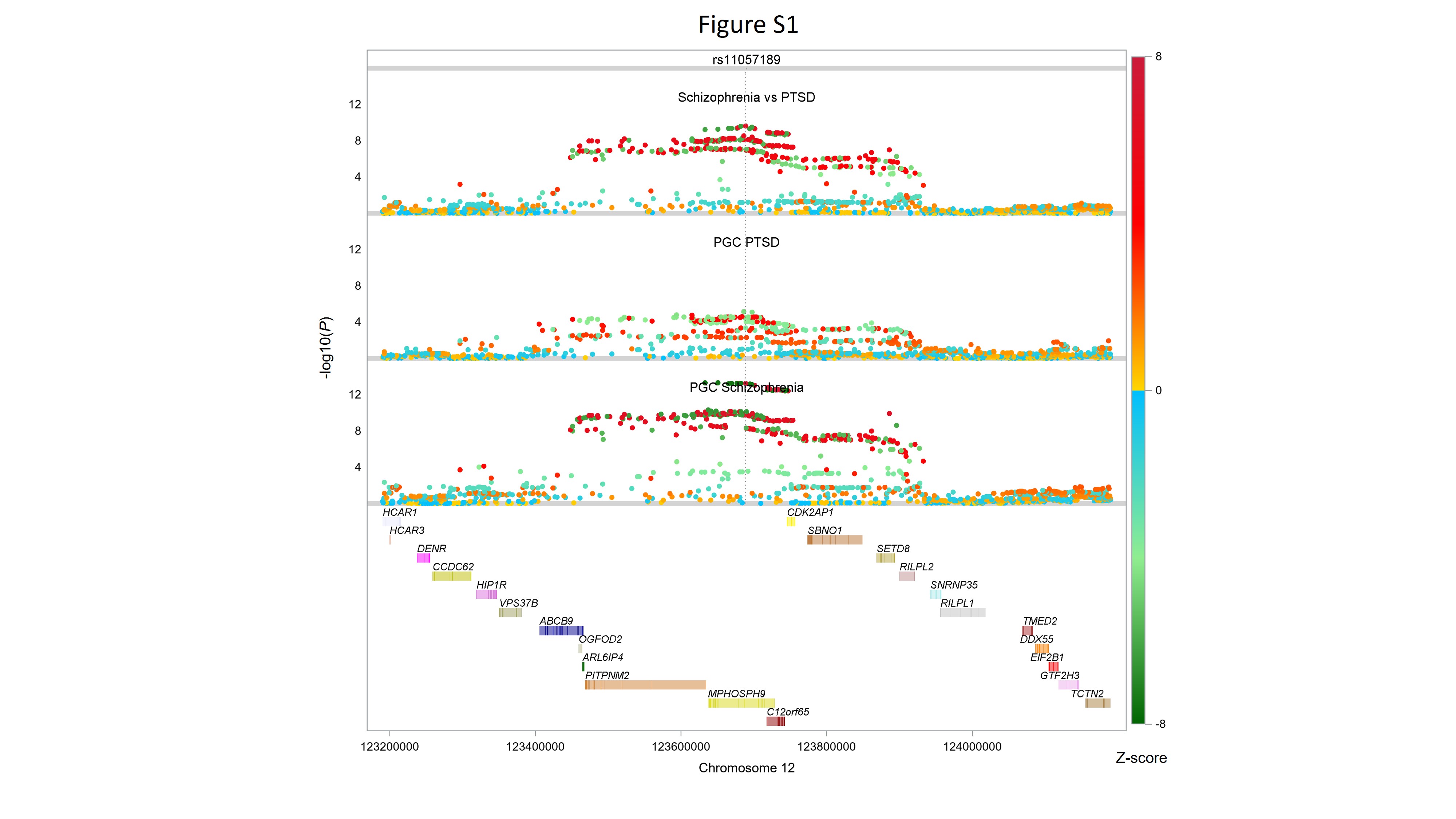

Supplement: Supplementary file 2 [file Image1.jpeg]

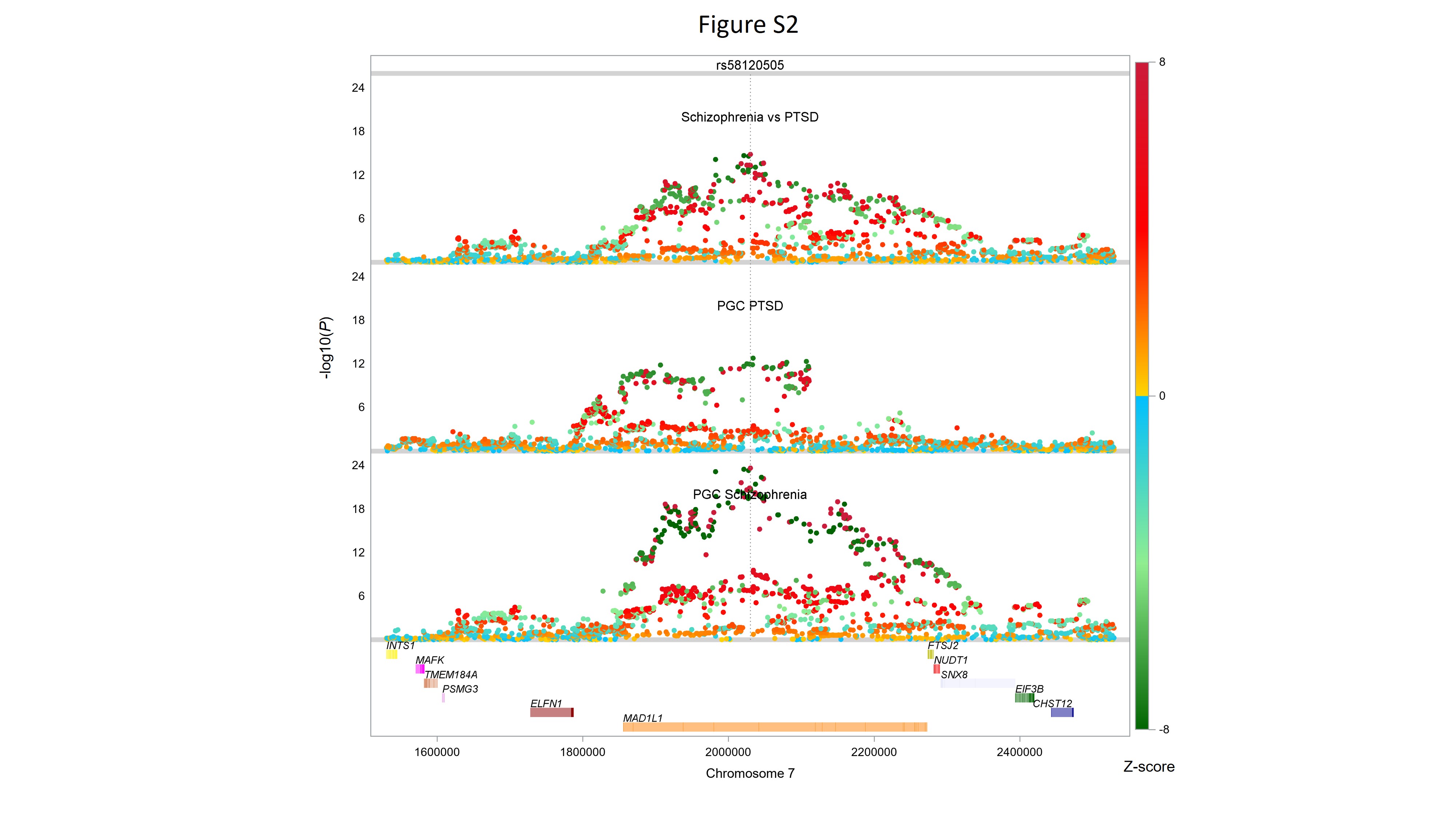

Supplement: Supplementary file 3 [file Image2.jpeg]

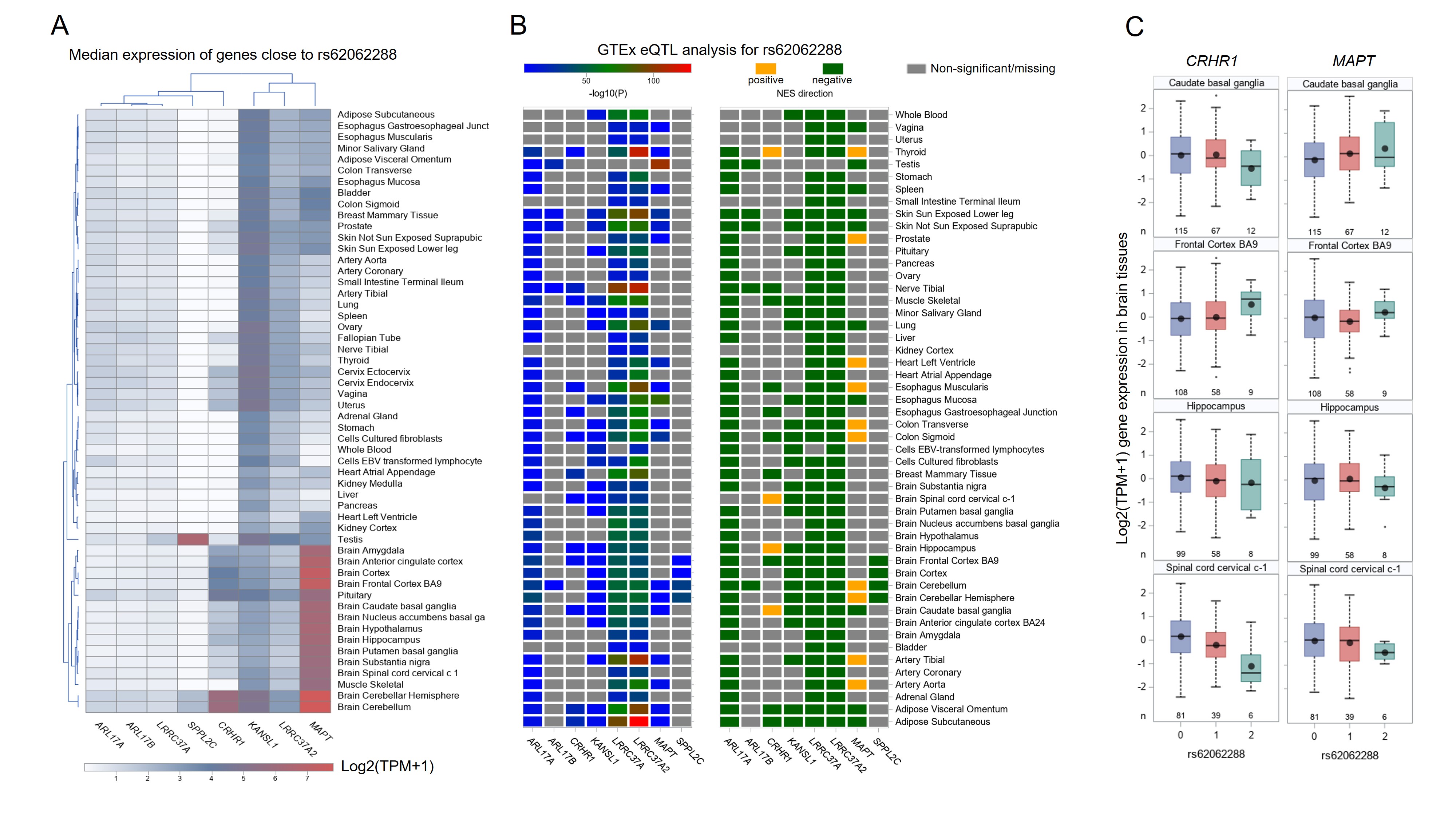

Supplement: Supplementary file 4 [file Image4.jpg]
